# Perceptions of Safety Without Experience: Age Differences in the Influence of Safety-Related Messages for AVs

**DOI:** 10.1093/geroni/igaf122.1996

**Published:** 2025-12-31

**Authors:** Ryan Best

**Affiliations:** West Virginia University, Morgantown, West Virginia, United States

## Abstract

The introduction of automated vehicle (AV) technologies to the roadways has the potential to improve road safety and reduce adverse outcomes. Benefits of AVs can play an outsized role for older adults considering age is commonly associated with a reduction in driving. As a cutting-edge technology where few have personal experience, individuals’ initial perceptions of the safety of AVs will likely stem from communications originating from several industry stakeholders, including AV companies themselves, outside regulators, and governing bodies. A survey instrument was developed to both implicitly and explicitly estimate how personal perceptions of AV safety are influenced by safety messages from different sources. Data was collected (n = 2,203) from the RAND American Life Panel, a nationally representative online probability sample. The implicit measure revealed that increasing age was generally associated with lower perceptions of AV safety, b=-.14, t=-6.48, p<.001. Compared to adults of higher ages, younger adults were particularly influenced by positive safety-related information provided by average AV crash rates, b=-.03, t=-4.03, p<.001, and official statements from AV companies, b=-.02, t=-3.90, p=.003. When measured explicitly, increased age was associated with a larger preference for safety-related messages from safety advocacy groups, ρ=.18, p<.001, and a decreased preference for information from average crash rates, rho=-.06, p=.005, and AV companies, rho=.14, p<.001. Results are discussed in terms of how safety related messages from various stakeholders might be utilized to affect perceptions of AVs across adulthood.

